# Detection of replicative integrity in small colonic biopsies using the BrdUrd comet assay

**DOI:** 10.1038/sj.bjc.6600836

**Published:** 2003-03-18

**Authors:** A P McGlynn, G R Wasson, S O'Reilly, V J McKelvey-Martin, H McNulty, J J Strain, G McKerr, F Mullan, N Mahmud, J Scott, D G Weir, C S Downes

**Affiliations:** 1 Cancer and Ageing Research Group, School of Biomedical Sciences, University of Ulster, Coleraine, County Londonderry, Northern Ireland, UK; 2 Department of Clinical Medicine, St James' Hospital, Dublin 8, Ireland; 3 Department of Biochemistry, Trinity College, Dublin 2, Ireland; 4 Causeway Trust, Coleraine, County Londonderry, Northern Ireland, UK

**Keywords:** comet assay, colon, DNA replication, bromodeoxyuridine

## Abstract

The alkaline single-cell gel electrophoresis or comet assay is a relatively simple method of measuring DNA single-strand breaks and alkali-labile sites in individual cells. Previously, we have used a combination of this with bromodeoxyuridine labelling of DNA and immunolocalisation of the BrdUrd to show that DNA replicative integrity can be assessed in single cultured cells. This study demonstrates the application of the technique to single cells derived from small human colonic biopsies isolated at routine endoscopy. A high level of reproducibility within replicate comet slides and between comet slides prepared from various colonic sites within a single patient is shown. Preliminary results demonstrate that defects in replication can be detected in tumour and premalignant colonic tissue adjacent to the tumour, suggesting that alterations in replicative integrity are an early event in neoplasia, appearing in premalignant mucosal cells. This development deems the BrdUrd comet assay suitable as an *ex vivo* molecular end point that can be measured easily in tissue collected by biopsy at routine colonic endoscopy. Thus, the BrdUrd comet assay has the potential to facilitate trial investigations of diet- or environment-related factors that may affect replicative integrity in the colon and provides a novel biomarker for colon carcinogenesis.

The comet assay is traditionally used to detect DNA damage (Ostling and Johanson, 1984; Fairbairn *et al*, 1995), and has been modified to detect more complex DNA lesions (Collins *et al*, 1993; Duthie and McMillan, 1997; McKelvey-Martin *et al*, 1998). We have described the combination of the alkaline comet method with pulse labelling of replicating DNA with the thymidine analogue bromodeoxyuridine (BrdUrd), and immunolocalisation of BrdUrd within the heads and tails of comets, so as to assess replicative integrity: that is, the efficiency of completion of DNA replication on a single cell basis (McGlynn *et al*, 1999). A label incorporated into DNA during a brief pulse of BrdUrd is initially close to strand discontinuities, and is seen as a fluorescently labelled comet tail upon electrophoresis. During pulse-chase experiments, as the replicating forks move away from the labelled regions, the label is incorporated into high molecular weight, continuous DNA and is observed as a comet head. The % BrdUrd label in the comet tail is therefore indicative of the proportion of recently replicated, imperfectly matured DNA in the nucleus. Using this BrdUrd comet assay, we were able to confirm that the known hypersensitivity of SVM84 cells to UV irradiation is because of a deficiency in UV postreplication repair (Pillidge *et al*, 1986; McGlynn *et al*, 1999). Although postreplication repair in this case, and in other mammalian cells, has been studied by biochemical methods (Brown, 1980; Clark and Hanawalt, 1984), these techniques are time-consuming, and require both radioactive labelling of DNA and the use of quite large cell populations (ca 10^5^ per data point) for which only an average figure can be obtained. The BrdUrd comet assay, on the other hand, can be simply and reproducibly performed using very small numbers of cells, and gives measurements on individual cells (McGlynn *et al*, 1999). We now describe the application of the BrdUrd comet assay to small numbers of epithelial cells derived from very small human biopsies.

Replication error positive (RER+) colorectal tumorigenesis occurs in individuals with hereditary nonpolyposis colorectal cancer (HNPCC), accounting for up to 5% of all colorectal cancer cases (Lynch *et al*, 1993) and in some sporadic tumours. How far replicative integrity and DNA maturation is defective in precancerous colon tissue, or varies between individuals, is unknown. The development of a method for the disaggregation of small human endoscopic biopsies, and the assessment of DNA metabolism at the single cell level, is of increasing importance. Here we describe such a method, together with pilot BrdUrd comet data from normal and diseased colonic mucosa. This development deems the BrdUrd comet assay suitable as a molecular end point that can be measured easily, hence facilitating trial investigations of diet- or environment-related factors affecting colorectal cancer.

## MATERIALS AND METHODS

### Tissue collection

Colonic biopsy specimens were obtained at endoscopy under a protocol approved by the ethics committees of the Federated Voluntary Dublin Hospitals. The study was approved by the ethics committee of the University of Ulster. Prior to endoscopy, patient details including age, sex and medical history were recorded (Table 1

**Table 1:** Details of patients specifically mentioned in the text including those diagnosed as histologically abnormal. Histological details of the study specimens are given together with the histological patient diagnosis following tissue analysis for clinical purposes

**Pt ID**	**Sex/age**	**FM Hx/diagnosis/current scope**	**Histology**	**Comet biopsy**
4	M/70	Rectal tumour	Adenoma moderate dysplasia, Dukes' B or	D9B1 tumour
			T3NOMX	D9B2 normal adjacent tissue
18	M/73	Diarrhoea	Routine bx – otherwise normal	Normal dsc
		Laxative atonic gut–some tissue changes		
23	M/70	Polyp r/v '98 Polyps in trans+divert dx	Tissue-trans-adenom-dysplastic-invasive	Normal dsc
25	No details	No details	No details	Site 1: normal asc
				Site 2: normal dsc
26	F/45	No details	No details	Site 1: normal asc
				Site 2: normal asc
28	M/67	Adenomatous mucosa polyps	Polyp-dsc-mod dys-adenomatous Dsc tissue–mild dysplasia	Normal dsc
29 Follow-up from 28	M/68	Polyp r/v This scope-polyp found at 50 cm (dsc/sig)	Routine bx –polyp base-normal histology	Normal sigmoid
35 Rpt of 23	M/71	Tubular adenoma in ileocaecal valve	Adenoma with low grade dysplasia	Site 1: adenoma
				Site 2: adjacent normal tissue
				Site 3: 10 cm asc colon – normal tissue
37	M/57	Normal	None done	Normal asc
				Normal trans
				Normal dsc
41	F/73	Fmhx crc Divert, HT	Polyp sigmoid-abnormal-tubular adenoma	Site 1: normal tissue distal sigmoid 10 cm
			+ mild dysplasia	Site 2: adenomatous rectosigmoid junction
				Site 3: normal tissue rectum 10 cm distal

Samples classified as normal are biopsies collected from subjects with no evidence of malignancy. Samples classified as abnormal are biopsies taken from polyp or adenoma tissue.

Asc=ascending, dsc=descending, caec=caecum, trans=transverse, r/v=review, divert=diverticulitis, dx=diagnosis, fm hx=family history, pr bleeding=peri-rectal bleeding, oeso=oesophagus, bx=biopsy, dys=dysplasia, crc=colorectal cancer.

).

### Isolation of epithelial cells from small colonic biopsies

Epithelial cell isolation was carried out using a method derived from that described by Meenan *et al* (1997). Briefly, two to four endoscopic biopsies from each area of interest were taken into calcium- and magnesium-free Hank's balanced salt solution (Life Technologies, Paisley, Scotland) supplemented with 0.3% bovine serum albumin (0.3%) and antibiotics (HBSSsup) and then transferred into HBSSsup containing 100 *μ*M BrdUrd at 37°C for 15 min. Subsequently, the specimens were processed for epithelial cell disintegration immediately or, in chase experiments, transferred into medium containing each of 200 *μ*M thymidine, 2′-deoxycytidine, 2′-deoxyguanosine and 2′-deoxyadenosine (Sigma, Dorset, UK). Biopsies were then transferred to fresh HBSSsup containing 0.75 mM dithiothreitol (Sigma, Dorset, UK) and were allowed to stand at room temperature for 3 h before being transferred to fresh HBSSsup containing 2 mM EDTA and placed on a rotating table, inclined at 45°, for 1 h at 37°C. The resulting suspension was washed and pelleted in HBSS, and after assessment of viability using ethidium bromide/acridine orange, cells were suspended stored in phosphate-buffered saline, at a concentration of 2 × 10^5^ cells ml^−1^, prior to comet slide preparation.

The purity of the cell preparation was confirmed using flow cytometry and a monoclonal antibody for the detection of epithelial cell antigen (Ber-Ep-4; Dako, Glostrup, Denmark) as described (Meenan *et al*, 1997) (data not shown).

### Cell culture

The established colonic cell line SW620 (Papadoupolous *et al*, 1995) was purchased from the ATCC (Rockville, MD, USA) and maintained in L-15 medium supplemented with 10% foetal bovine serum and antibiotics in a humidified incubator at 37°C, 5% CO_2_. Prior to BrdUrd pulsing (20 *μ*M for 20 mins), cells were plated at a density of 5 × 10^4^ cells per 60 mm dish for 48 h. For chase experiments, cells labelled with BrdUrd were incubated for 1 h in complete medium containing each of 200 *μ*M thymidine, 2′-deoxycytidine, 2′-deoxyguanosine and 2′-deoxyadenosine (Sigma, Dorset, UK). BrdUrd incorporated into DNA during a brief pulse is initially close to strand discontinuities in the comet tail, but during pulse-chase experiments in cells capable of normal DNA maturation, the BrdUrd is incorporated into high molecular weight, continuous DNA represented by the comet head.

### Comet slide preparation and BrdUrd comet assay

The BrdUrd comet assay was performed as previously described (McGlynn *et al*, 1999). Briefly, cells were washed in cold PBS, and the cell pellet was resuspended in 100 *μ*l of 0.75% low melting point (LMP) agarose at 37°C. The cell suspension was spread onto a standard comet assay slide (Singh *et al*, 1988) and lysed in a neutral lysis buffer containing 0.1% LiDS, pH 8.0, and 0.03 mg ml^−1^ proteinase K, at 37°C overnight followed by alkaline lysis, pH 10 for 1 h at 4°C.

DNA unwinding (40 min) and electrophoresis (20 min at 25 V and 300 mA) were carried out under alkaline conditions (0.3 M NaOH, 1 mM EDTA, pH 13). Following electrophoresis, the gels were neutralised (0.4 M Tris) and washed with PBS prior to immunostaining. The gels were incubated with 25 *μ*l per gel of mouse monoclonal anti-BrdUrd (10 *μ*g ml^−1^; BO Biosciences, Oxford, UK) in the dark at room temperature for 1 h. The primary antibody was gently washed off with three changes of PBS and one wash with PBS/0.1% BSA, before the addition of 25 *μ*l per gel of secondary antibody (5 *μ*g ml^−1^ sheep anti-mouse IgG, fluorescein conjugated: Bioscience, Oxford, UK), which was incubated and washed off as before. The gels were then counterstained with 25 *μ*l of propidium iodide (0.75 *μ*g ml^−1^; Sigma, Dorset, UK) and covered with a coverslip (22 × 50 mm^2^) for image analysis.

### Comet analysis

**Figure 1:**
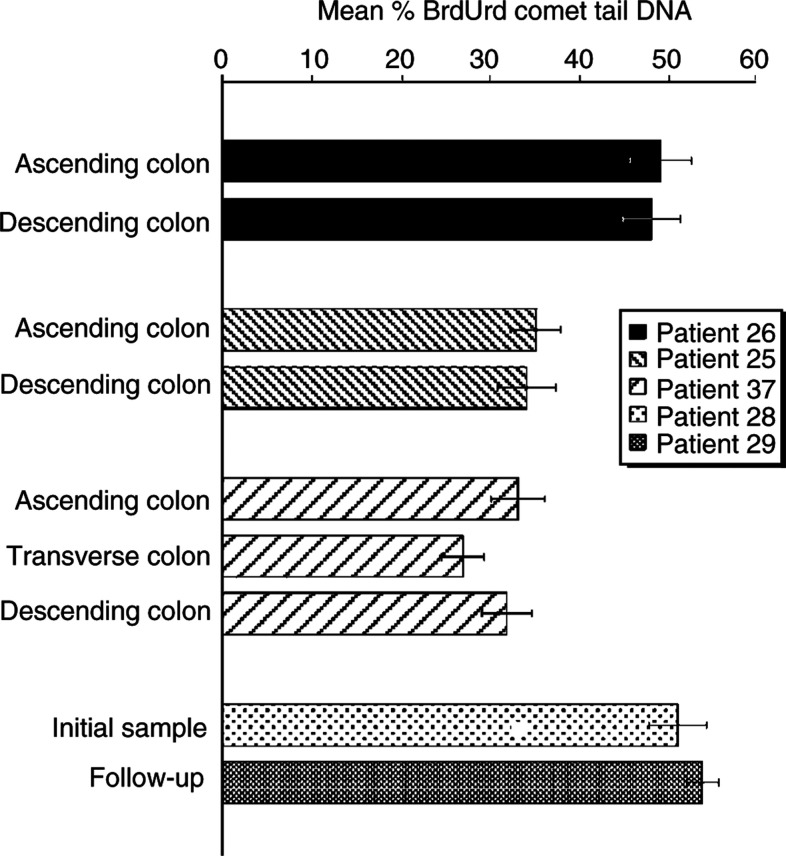
Reproducible BrdUrd comet measurements from cells derived from human colonic biopsies. Results are presented as mean % BrdUrd comet tail DNA with patient ID numbers given in the legend. Representative examples are shown demonstrating the reproducibility of adjacent sites in the ascending colon, patient ID 26, and of different sites in the colon; ascending *vs* descending, patient ID 25; ascending *vs* transverse *vs* descending, patient ID 37. Follow-up data for one patient is also presented comparing the initial sample (patient ID 28) with a sample taken 4 months later (patient ID 29).

For biopsy specimens, comet slides were analysed using a single-blind approach in that patient details were not made available until comet analysis was completed. Comet analysis was carried out as previously described (McGlynn *et al*, 1999) using a final magnification of × 400 (Nikon × 40 Fluor lens) and Komet 4.0 software (Kinetic Imaging Ltd, Liverpool, UK). The % DNA in the comet tail for each cell was measured. Results were expressed as mean % comet tail DNA in 25 cells on each of duplicate comet slides. Data are collected as the mean and standard deviation of the 50 comets scored per individual patient (as shown in Figure 1

). The variance or dispersion from the mean of % tail DNA of 50 comet measurements from each duplicate pair of slides was also noted.

## RESULTS

### Mucosal epithelial cell isolation

Using the ion-chelation method described, an epithelial cell population of greater than 95% purity was obtained, as assessed by flow cytometry using epithelial cell antigen positive expression. Contaminating lymphocytes could be excluded on the basis of size during comet analysis.

Cell yield ranged from 1.0 to 2.0 × 10^5^ cells per single biopsy. Cell viability, as assessed using acridine orange and ethidium bromide, ranged from 60 to 80%, and was found to be much greater than that found using enzymatic digestion, either alone or in combination with ion chelation. Specimens from the ascending colon and caecum produced higher cell yields and greater viability.

### Highly reproducible comet data demonstrates little variation of replicative integrity among normal subjects

Inter- and intrapatient reproducibility of the BrdUrd comet assay was assessed. Comet measurements on duplicate endoscopic biopsies, which were collected from up to three different colorectal sites in each patient (*n*=36 sites), showed no significant difference between sites, demonstrating the reproducibility of the BrdUrd comet assay applied to colonic biopsy cells (Figure 1). Furthermore, a sequential follow-up specimen was analysed from one patient undergoing adenomatous polyp review. Upon first analysis, the patient displayed dysplastic polyps and had a mean % comet tail of 50±11 in comets derived from normal descending colon, upon reanalysis 4 months later, the patient was again diagnosed with adenomatous polyps and had a mean % comet tail of 53±9 in comets derived from histologically normal sigmoid tissue (Figure 1). This indicates that follow-up clinical studies using the BrdUrd-comet assay may be possible. Further statistical analysis is necessary to confirm these observations. No significant difference in mean % comet tail was observed between duplicate comet slides (results not shown).

**Figure 2:**
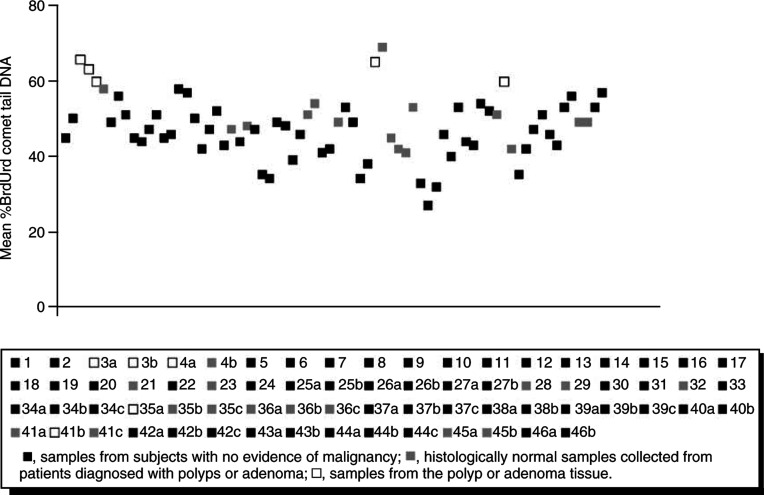
BrdUrd comet analysis of colonic mucosal cells disaggregated from human colonic biopsies. Results presented as a scatterplot of mean % BrdUrd comet tail DNA. Patient ID numbers (*n*=46) are given in the legend and correspond to patient details in Table 1. Multiple samples from the same patient are labelled a, b and c.

A total of 52 biopsies were collected from 35 patients who displayed no evidence of malignancy (normals) upon endoscopic investigation. Little variation in mean % BrdUrd comet tail (mean comet tail of 52 biopsies 46, s.d. 7.1) was observed among this sample group (Figure 2

).

### Aberrant BrdUrd comets of colon cells from subjects with colonic polyps or adenoma

Pilot data revealed that biopsies of polyp or tumour tissue have a significantly higher mean % comet tail DNA (mean of 63±2.7, collective comet data for *n*=4, *P*<0.001) compared to those from tissue collected from normal subjects (collective comet mean 46±7.1, *n*=52 normal samples) (Figure 2). This indicates a detectable deficiency in DNA replication in these samples.

The abnormal samples included tumour tissue from a Dukes stage B invasive rectal adenoma (patient ID 4), polyp tissue from a patient diagnosed with tubulovillus adenoma with mild dysplasia (patient ID 3), adenomatous tissue from a tubular adenoma in the ileocaecal valve (patient ID 35) and adenomatous tissue from a tubular adenoma in the rectosigmoid junction (patient ID 41). Histologically normal tissue from patients with polyps/adenoma (*n*=15) also showed a trend towards an elevated mean % comet tail DNA compared to normals in most cases (collective comet mean of 50±7.1, *n*=15), although these results did not reach significance.

### BrdUrd comet analysis of multiple samples from patients with malignancy

**Figure 3:**
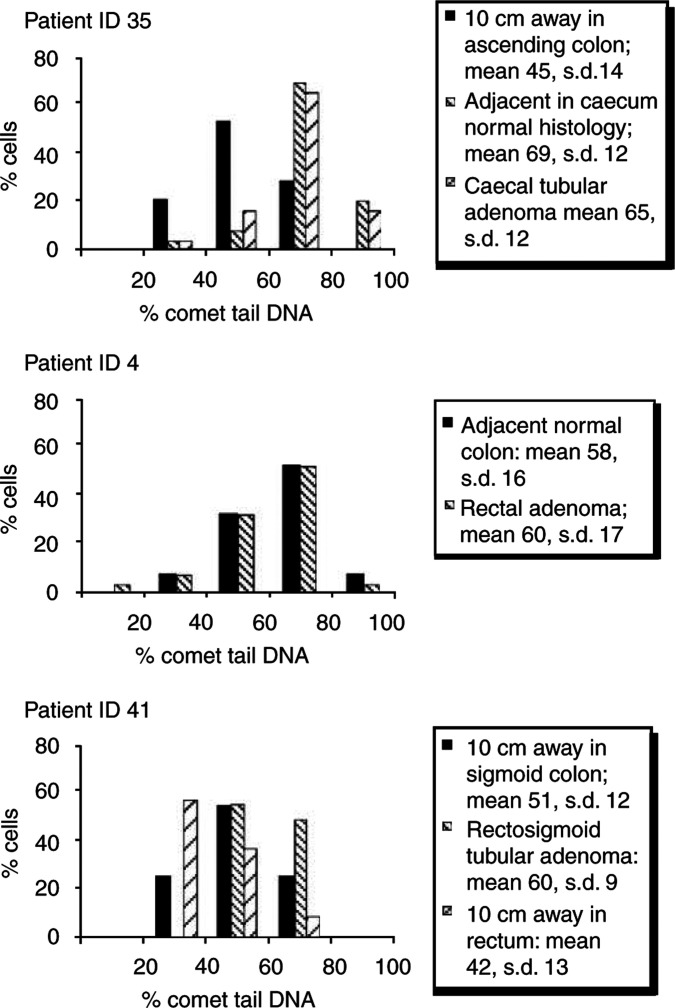
Evidence of aberrant BrdUrd comet analysis in tumour and adjacent normal colon but not in distal normal colon. Biopsies were collected from multiple sites – from the adenoma, histologically normal-appearing mucosa adjacent to the adenoma and histologically normal-appearing mucosa 10 cm away from the adenoma – and disaggregated before pulsing with BrdUrd for 15 min and processing for the BrdUrd comet assay. Results are expressed as frequency distribution plots of BrdUrd percentage of tail DNA and mean + standard deviation, s.d.

Multiple biopsies were collected from various sites within the colon of patients diagnosed with malignancy in order to assess the degree to which the deficiency in DNA replication found in histologically abnormal samples extends into the histologically normal tissue surrounding a polyp/adenoma. In the first patient (patient ID 35), biopsies were collected from a tubular adenoma of the caecum and from histologically normal tissue both adjacent to and 10 cm away from the adenoma. Cells from both the adenomatous tissue and the adjacent histologically normal tissue showed elevated comet measurements as distinct from the tissue 10 cm away, which showed normal comet measurements (Figure 3

, patient ID 35).

**Figure 4:**
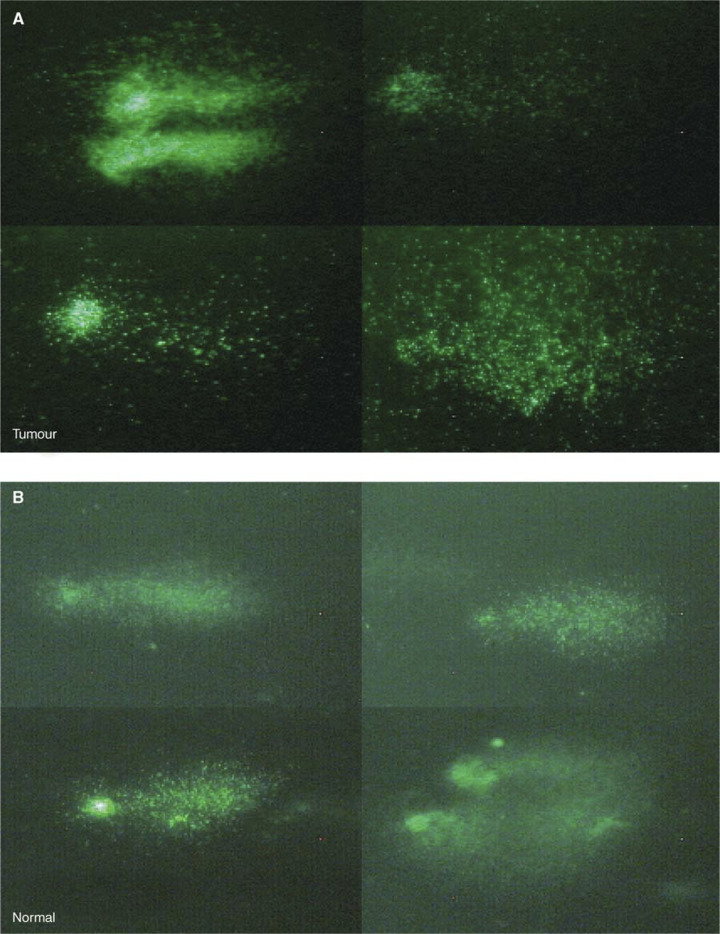
Variable appearance of BrdUrd comets derived from rectal tumour biopsy compared with adjacent colon tissue. BrdUrd comet images from a single slide prepared from a rectal tumour (**A**) compared with those from a single slide prepared from an adjacent normal tissue (**B**). The tumour-derived comets show increased variation in comet appearance in relation to head/tail DNA content, tail length and degree of BrdUrd incorporation as compared with the more homogeneous normal comets.

Similar results were found in a second subject (Figure 3, patient ID 4), where the normal adjacent tissue showed similar elevated comets to that of the rectal adenomatous tumour. Interestingly, the rectal tumour tissue displayed both an elevated mean % comet tail DNA and a high variance in % tail DNA (variance=360 compared to overall average variance for normal samples of 219±88, *n*=52 samples). On the other hand, while the histologically normal tissue adjacent to the tumour showed a mean % comet tail DNA similar to that of the tumour, it did not display observable comet variation (variance=232). This variability in comet formation was seen in head/tail DNA content, tail length and degree of BrdUrd incorporation, which is possibly indicative of the high degree of tumour anaplasia (Figure 4

, patient ID 4).

In a third patient diagnosed with tubular adenoma and mild dysplasia at the rectosigmoid junction (Figure 3, patient ID 41), samples were collected from the adenomatous tissue and from two sites 10 cm distal to the adenoma in the sigmoid colon and rectum. As with the data from patient ID 35, the adenomatous tissue displayed an elevated % comet tail DNA, but the two sites distal from the adenoma showed reduced comet tails (Figure 3, patient ID 41).

### Defective DNA replicative integrity is detectable using BrdUrd pulse-chase in colonic cells

**Figure 5:**
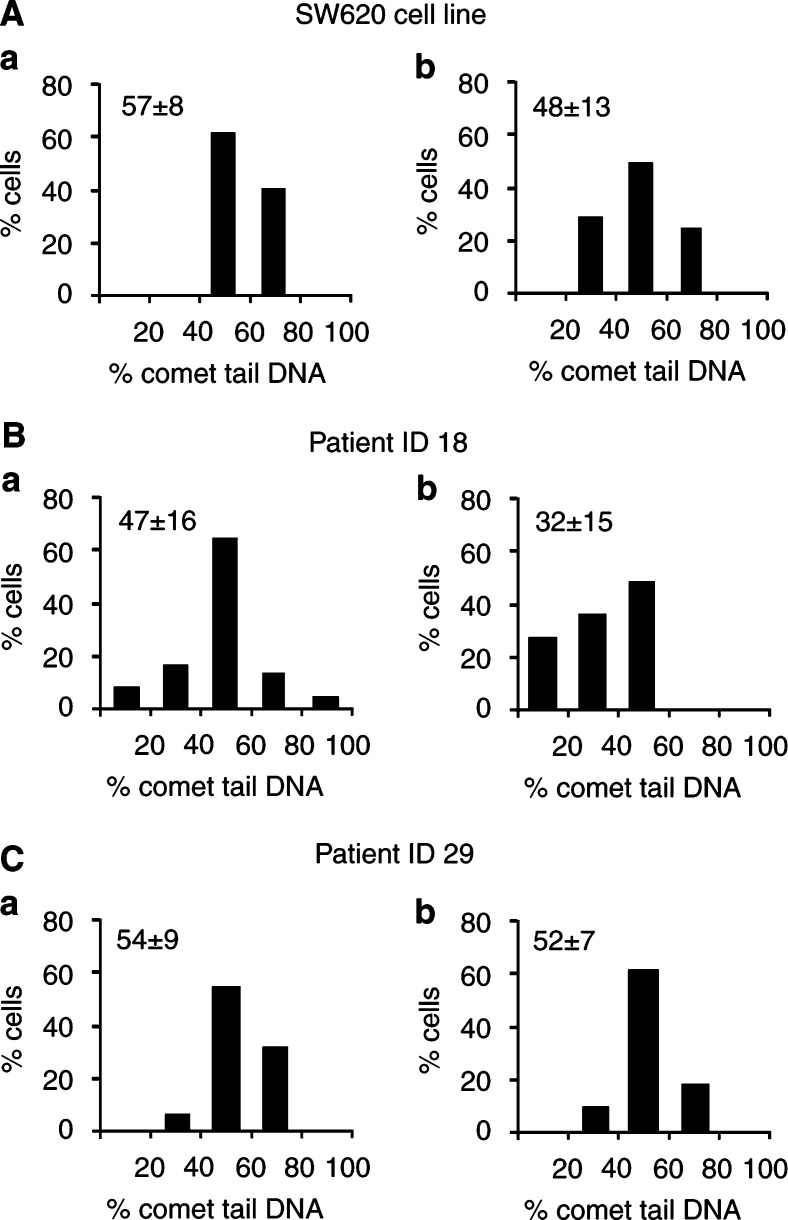
BrdUrd pulse-chase in colonic cells reveals inherent defects in DNA replicative integrity. BrdUrd comet analysis of colonic mucosal cells disaggregated from human colonic biopsies and control cultured cell lines. Cells were given a pulse of BrdUrd and either processed immediately for the BrdUrd comet assay (**A**) or chased with 200 *μ*M of all four deoxynucleotides for 1 h prior to comet processing. (**B**) SW620 colon cell line, (**C**) normal colonic mucosa (patient ID 18) and mucosal cells from a patient presenting with adenomatous polyps and moderate dysplasia (patient ID 29). Results are expressed as frequency distribution plots of BrdUrd percentage of tail DNA labelled as BrdUrd pulse alone or BrdUrd pulse with chase.

For pulse-chase experiments, entire mucosal biopsies were transferred to chase medium following BrdUrd labelling. To calibrate the assessment of DNA replicative integrity using BrdUrd comet pulse-chase experiments, a transformed colonic cell line was used (SW620; Figure 5

) which showed some DNA maturation after the chase period. Mucosal cells derived from a normal biopsy (patient ID 18) displayed more proficient DNA maturation after a chase period of only 1 h (*P*<0.01). However, mucosal cells from a single patient presenting with adenomatous polyps and moderate dysplasia (patient ID 29) demonstrated a significant delay in DNA maturation compared to patient ID 18 (*P*>0.05, *t*-test), evidenced by a failure of the comet tails to move into the head (Figure 5).

## DISCUSSION

There is substantial evidence that the formation of a tumour is preceded by a series of somatically inherited changes, that is mutations or epigenetic events such as DNA methylation (Baylin, 1997; Bernstein *et al*, 2000). These changes lead to abnormalities of cell growth such as increased cell proliferation and genomic instability. The identification of these early molecular defects or markers in cells with normal morphology is important for identifying subjects at high risk of developing cancer. These molecular markers may also be used as biomarkers in studies to identify risk factors for carcinogenesis. We have recently developed a novel assay for the detection of DNA synthesis and maturation in single cells, which we have calibrated with cell lines grown in culture (McGlynn *et al*, 1999).

Molecular studies of colon carcinogenesis have been performed using measures of proliferative compartments of colonic crypts (Terpstra *et al*, 1987; de Leon *et al*, 1988), ploidy assessment of colonic carcinomas (Jarvis *et al*, 1987; Cianchi *et al*, 1999) and cell cycle kinetics of colonic cells *in vivo* using flow cytometry (Michel *et al*, 2000). Each of these methods indicates that faulty cell division in the colon occurs in regions adjacent to polyps or carcinomas, and is therefore taken to be responsible for the progression from early neoplastic lesions to the development of an adenomatous polyp to an invasive carcinoma. (Strictly speaking, these data are equally compatible with the hypothesis that cells in the later stages of carcinogenesis exert a malign influence on the surrounding, normal tissues, an issue discussed below.)

The novel BrdUrd comet is entirely separate from DNA methodologies measuring BrdUrd-labelled DNA with flow cytometry, in that it measures precisely the efficiency of DNA maturation in colonic cells, whether from histologically normal or abnormal tissue. An increase in comet tail length is indicative of a delay in DNA maturation, because of stalling of the replication fork at DNA lesions or defects in postreplicative repair.

In the present study, the BrdUrd comet assay was successfully applied to single colonic epithelial cells derived from disaggregated human biopsies; we show that defective DNA replication may be suitable as a molecular marker for early stages of colon carcinogenesis.

The comet assay was originally designed to detect DNA damage; therefore the choice of method for tissue disaggregation to maintain cell viability and intact DNA is imperative. In this study, the ion-chelation method of biopsy disaggregation proved superior to enzymatic digestion on the basis of combined cell yield and viability (two-fold in each case). Minimal DNA damage was induced in cells isolated in this way compared with enzymatic digestion as determined by propidium-iodide-labelled comet tails. This ion-chelation method was adapted from that described in detail by Meenan *et al* (1997) and designed to isolate epithelial colonocytes from the heterogeneous population of cells of the intestinal mucosa. We have demonstrated that the BrdUrd comet assay is suitable for the measurement of DNA maturation and integrity in very small numbers of primary epithelial cells derived from biopsy specimens of human colonic mucosa.

By exposing the cells to a short pulse of BrdUrd alone and measurement of BrdUrd comet tails, much information regarding the status of DNA maturation can be gained. The assay was shown to be reproducible in that duplicate specimens of colonic mucosa gave very similar results regardless of the site of biopsy. This was shown to be the case in both normal mucosa and in biopsied adenomatous polyp tissue (Figure 1). Furthermore, results from a cohort of histologically normal colonic tissue samples showed a low level of variability within this population (Figure 2). On the other hand, this study shows that it is possible to detect elevated BrdUrd comets from histologically abnormal tissue (rectal tumour, polyp tissue from a patient with tubulovillus adenoma and tubular adenomas) when compared with that from normal mucosa, by an increase in % tail DNA (*P*<0.001), indicative of stalled maturation (Figure 2). This may be because of a high level of intrinsic DNA damage and/or defective postreplication repair machinery in the tumour cells (Johnson *et al*, 1994).

Interestingly, increased BrdUrd comet tails were also found in samples of histologically normal tissue from patients with an abnormal diagnosis, although these data did not reach statistical significance. This would be compatible with a decrease in replicative integrity in histologically normal tissue with an underlying molecular defect and progression towards malignancy, or, as mentioned above, with a malign effect of tumour cells on their neighbours. This is supported by data from patients IDs 23 and 35, diagnosed with tubular adenoma (Figure 3), where there is an increase in the mean % BrdUrd comet tail of histologically normal tissue upon follow-up investigation. In order to investigate further the role played by replicative status in tumour formation and progression in the colon, multiple biopsies were examined from neoplastic tissue, histologically normal tissue adjacent to the tumour and normal tissue distal to the tumour from patient IDs 4, 35 and 41. Interestingly, elevated BrdUrd comets were found, not only in the tumour but also in the adjacent tissue, whereas normal levels were found in the tissue 10 cm distal to the tumour (Figure 3). Although the Dukes' stage B rectal tumour and adjacent tissue of patient ID 4 both displayed elevated BrdUrd comets, a high degree of variability in comet appearance was associated with the tumour only, possibly reflecting the high degree of anaplasia of the tumour, not yet seen in the adjacent tissue (Figure 4).

Taken together, these results support the hypothesis that the underlying defective replication exists in histologically normal colonic tissue, which may predispose to neoplasia, or, conversely, that neoplastic tissues malignly affect normal neighbouring cells. Further, these results are in agreement with previous findings showing changes in cell proliferation in normal mucosa of colorectal cancer patients (Terpstra *et al*, 1987) and may indicate that aberrant replicative integrity is an early event in neoplasia in premalignant cells. It is possible that the adenomas in patient IDs 35 and 41 developed in an area of the colon with replicative abnormalities but that defective replication was not a colon-wide phenomenon.

A further development of the BrdUrd comet assay is the introduction of a pulse-chase step, to allow the maturation of DNA over time (McGlynn *et al*, 1999). A reduction in tail DNA was evident in normal mucosal epithelial cells in a 1-h chase period, which was more efficient than in either epithelial cells taken from a dysplastic mucosa (patient ID 29) or in a cultivated colon tumour line (Figure 5). These results further support the ability of the BrdUrd comet assay to assess DNA maturation and determine replicative integrity in human colonic biopsies.

The present data on the application of the BrdUrd comet assay demonstrate that DNA maturation and postreplicative repair can now be determined in small numbers of cells derived from human colonic biopsies taken during routine endoscopy. Studies to investigate the effects of diet and other factors on DNA replication in the colon, using this assay, are under way.
